# Bcr-abl regulates Stat5 through Shp2, the interferon consensus sequence binding protein (Icsbp/Irf8), growth arrest specific 2 (Gas2) and calpain

**DOI:** 10.18632/oncotarget.12749

**Published:** 2016-10-19

**Authors:** Elizabeth E. Hjort, Weiqi Huang, Liping Hu, Elizabeth A. Eklund

**Affiliations:** ^1^ The Feinberg School at Northwestern University, Chicago, IL, USA; ^2^ Jesse Brown VA Medical Center, Chicago, IL, USA

**Keywords:** leukemia, calpain, transcription factor, myeloid, phosphatase

## Abstract

Icsbp/Irf8 is an interferon regulatory transcription factor that functions as a suppressor of myeloid leukemias. Consistent with this activity, Icsbp represses a set of genes encoding proteins that promote cell proliferation/survival. One such gene encodes Gas2, a calpain inhibitor. We previously found that increased Gas2-expression in Bcr-abl^+^ cells stabilized βcatenin; a Calpain substrate. This was of interest, because βcatenin contributes to disease progression in chronic myeloid leukemia (CML). Calpain has additional substrates implicated in leukemogenesis, including Stat5. In the current study, we hypothesized that Stat5 activity in CML is regulated by Gas2/Calpain. We found that Bcr-abl-induced, Shp2-dependent dephosphorylation of Icsbp impaired repression of *GAS2* by this transcription factor. The consequent decrease in Calpain activity stabilized Stat5 protein; increasing the absolute abundance of both phospho and total Stat5. This enhanced repression of the *IRF8* promoter by Stat5 in a manner dependent on Icsbp, Gas2 and Calpain, but not Stat5 tyrosine phosphorylation. During normal myelopoiesis, increased expression and phosphorylation of Icsbp inhibits Calpain. In contrast, constitutive activation of Shp2 in Bcr-abl^+^ cells impairs regulation of Gas2/Calpain by Icsbp, aberrantly stabilizing Stat5 and enhancing *IRF8* repression. This novel feedback mechanism enhances leukemogenesis by increasing Stat5 and decreasing Icsbp. Bcr-abl targeted tyrosine kinase inhibitors (TKIs) provide long term disease control, but CML is not cured by these agents. Our studies suggest targeting Calpain might be a rational therapeutic approach to decrease persistent leukemia stem cells (LSCs) during TKI-treatment.

## INTRODUCTION

Chronic myeloid leukemia (CML) is characterized by reciprocal translocations involving chromosomes 9 and 22 that generate the tyrosine kinase oncogene, Bcr-abl [1]. Clinical outcomes in CML were revolutionized by development of Bcr-abl specific tyrosine kinase inhibitors (TKIs), but most patients are not cured by these agents [2–5]. Specifically, in several clinical trials, the majority of CML subjects relapsed upon discontinuation of TKI treatment, including those with an optimal clinical response [6–8]. Most subjects with relapse obtained a second remission with TKI treatment, but the time to second remission was longer than to first remission. These studies indicate persistence, and perhaps expansion, of a subset of leukemia stem cells (LSCs) during TKI treatment. Identifying molecular mechanisms involved in LSC persistence may suggest molecular therapeutic targets to augment TKI treatment in CML. We hypothesize that events regulated by the leukemia suppressor Icsbp (encoded by the *IRF8* gene) are potential candidates for therapeutic targeting.

Icsbp is expressed at low levels in CD34^+^ bone marrow cells from CML subjects in comparison to normal CD34^+^ cells [9, 10]. Icsbp expression rises in remission due to TKI or interferon, falls with emergence of drug resistance, and is lowest in blast crisis (BC). Several murine models suggested a functional role for Icsbp as a CML suppressor. In one such study, mice were transplanted with bone marrow expressing the Bcr-abl oncogene, with or without re-expression of Icsbp [11]. Development of CML was delayed in mice with bone marrow expressing Bcr-abl + Icsbp in comparison to Bcr-abl alone [11]. In another murine model, disruption of the *IRF8* gene led to granulocytosis that progressed to acute myeloid leukemia (i.e. BC) with time [12, 13]. These murine models phenocopied human CML and exhibited CML-like alterations in gene expression [9, 14-16].

Although Icsbp was initially described as a regulator of immune effector genes, it also regulates cell proliferation and/or survival through target genes encoding Nf1, Bcl2, Klf4, Fap1 and Gas2 [14-19]. Fap1 (Fas associated phosphatase 1) inactivates Fas and Gsk3β [15, 20-22]. During normal myelopoiesis, repression of the Fap1 gene (*PTPN13*) by Icsbp increases sensitivity to Fas-induced apoptosis and facilitates proteasomal degradation of βcatenin as differentiation proceeds [15, 20]. We found that Fap1 inhibition in a murine model of CML delayed emergence of TKI resistance and decreased LSC persistence *in vivo*, suggesting relevance of Icsbp/Fap1 regulated events [23].

Gas2 binds to Calpain and inhibits its serine protease activity [24, 25]. We found that repression of *GAS2* transcription by Icsbp increased Calpain activity during differentiation of myeloid progenitor cells [16]. Since βcatenin is a Calpain substrate, this decreased expression of βcatenin target genes such as c-Myc, Survivin and Cyclin D1 [16]. Calpain has other substrates of potential significance to the pathogenesis of CML, including Stat5 [26]. In the current work, we explore the possibility that Icsbp regulates Stat5 through Gas2/Calpain. The goal of these studies was to investigate Calpain activity as a potential therapeutic target to augment TKIs and decrease LSC persistence in CML.

Activity of Icsbp is regulated at transcriptional and post-translational levels. In HSC and early progenitor cells, Icsbp is maintained in a non-tyrosine phosphorylated state by Shp2-protein tyrosine phosphatase (PTP) [17]. As myelopoiesis proceeds, Icsbp is increasingly tyrosine phosphorylated in response to cytokines in a manner that depends on Jak2-activation and Shp2-inactivation [13, 27]. We found that expression of a leukemia associated, constitutively active mutant of Shp2 sustained Icsbp in the non-phosphorylated state, despite cytokine stimulation, and accelerated progression to BC in Icsbp^+/−^ mice [13]. In addition to mutations in the Shp2 gene, other leukemia associated mutations have been described that result in constitutive activity of Shp2, including internal tandem duplication of *FLT3* in acute myeloid leukemia (AML) and expression of Bcr-abl in CML [28, 29]. Since Icsbp tyrosine phosphorylation facilitates repression of the Gas2 and Fap1 genes, either decreased expression or impaired phosphorylation of Icsbp would result in progenitor expansion [16, 19, 30].

Since Stat5 is a Calpain substrate, decreased activity of Icsbp in CML might stabilize Stat5 protein. We considered the possibility that cross regulation of these two transcription factors, one a leukemia facilitator and the other a leukemia suppressor, contributes to the pathogenesis of CML. However, mechanisms modulating expression of Icsbp during myelopoiesis or leukemogenesis are ill defined. Other investigators described repression of the *Irf8* promoter by Stat5 during dendritic cell differentiation [31]. This was somewhat unexpected, because monocytes from Icsbp^−/−^ mice were unable to differentiate to dendritic cells [12]. In another study, Stat5 decreased Icsbp expression in K562 cells; a line derived from a patient with erythroid blast crisis of CML [32]. However, relevance of these observations to the biology of chronic phase (CP) CML is complicated by differential Stat usage in erythroid versus myeloid lineages, and the differential biology of CML-CP versus BC.

Our studies investigate a novel, Calpain-dependent mechanism resulting in cross regulation between Icsbp and Stat5 in Bcr-abl^+^ myeloid progenitor cells that promotes leukemogenesis. The goal of these studies is to determine if Icsbp modulates Stat5 activity in CP-CML through Gas2 and Calpain. If Calpain activation both increases Icsbp (a leukemia suppressor) and decreases Stat5 (a leukemia promoter) in CML-LSCs, this might be a rationale therapeutic approach to enhance LSC-targeting during TKI treatment.

## RESULTS

### Bcr-abl decreases Calpain activity by activating Shp2, resulting in decreased GAS2 repression by Icsbp

To investigate the role of Calpain in the pathogenesis of CML, we first determined the mechanism for increased expression of the calpain inhibitor Gas2 in Bcr-abl expressing cells. In previous studies, we found repression of the *GAS2* promoter by Icsbp was enhanced by phosphorylation of tyrosine residues in the Icsbp-DNA-binding domain [16]. Since Bcr-abl activates Shp2 and Icsbp is a Shp2 substrate [17, 29], we hypothesized activation of Shp2 by Bcr-abl would de-phosphorylate Icsbp and increase *GAS2* transcription. To test this hypothesis, we transfected U937 myeloid leukemia cells with a 130 bp *GAS2* promoter/reporter construct (including an Icsbp-binding cis element) and various combinations of vectors to express Bcr-abl, Icsbp, a tyrosine mutant form of Icsbp (Y-mut Icsbp), or a dominant negative form of Shp2 (C463S) (or relevant control vectors).

**Figure 1:**
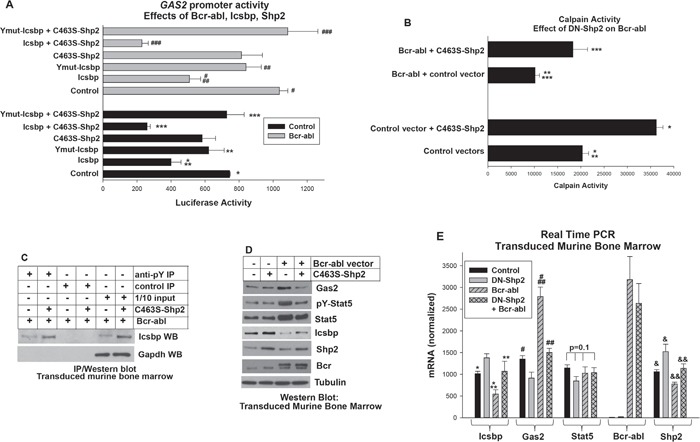
Bcr-abl inhibited Calpain by decreasing Gas2 expression through dephosphorylation of Icsbp by Shp2 **A.** Activation of Shp2 by Bcr-abl impaired repression of the *GAS2* promoter by Icsbp. U937 cells were transfected with a reporter vector with 130 bp of the *GAS2* promoter (with an Icsbp-binding cis element). Cells were co-transfected with a Bcr-abl expression vector (or control) with or without vectors to express C463S-Shp2 (dominant negative), Icsbp, or a form of Icsbp with mutation of tyrosine residues in the DNA-binding domain (Ymut-Icsbp). Statistically significant differences indicated by *, **, ***, #, ## or ###. **B.** Decreased calpain activity in Bcr-abl^+^ cells was reversed by inhibition of Shp2. Murine bone marrow myeloid progenitor cells were transduced with a retroviral vectors to express Bcr-abl (or control) with or without C463S-Shp2 and assayed for Calpain activity. Statistically significant differences indicated by *, **, or ***. **C.** Tyrosine phosphorylation of Icsbp was increased by inhibition of Shp2 activity in Bcr-abl^+^ cells. Cells lysates from the transduced cells described above were immuno-precipitated with an anti-phospho tyrosine antibody (or irrelevant control antibody) and Western blots serially probed with an antibody to Icsbp and Gapdh (to control for loading). **D.** Inhibition of Shp2 activity in Bcr-abl^+^ cells decreased Gas2 and Stat5 protein, but increased Icsbp protein. Western blots of lysates from the transduced cells were serially probed with antibodies as indicated. **E.** Inhibition of Shp2 activity in Bcr-abl^+^ myeloid progenitor cells decreased Gas2 mRNA, increased Icsbp mRNA, but did not alter Stat5 mRNA. Gene expression in transduced murine bone marrow cells was analyzed by real time PCR. Statistically significant differences indicated by *, **, #, ##, & or &&. For all of these studies, p<0.02 was considered statistically significant.

We found significantly more *GAS2* promoter activity in Bcr-abl^+^ transfectants in comparison to control (p<0.0001, n=4). This Bcr-abl effect was reversed by overexpression of Icsbp (p<0.001, n=4 for Bcr-abl with versus without Icsbp (Figure 1A). To determine if activation of Shp2 by Bcr-abl impaired repression of the *GAS2* promoter by Icsbp, we co-transfected U937 cells with *GAS2* promoter-reporter construct, a vector to overexpress Icsbp, and vectors to express Bcr-abl, C463S-Shp2, or both. Expressing a dominant negative form of Shp2 significantly increased repression of the *GAS2* promoter by overexpressed Icsbp, with or without Bcr-abl (p<0.001, n=4 for Icsbp with versus without C463S-Shp2) (Figure 1A).

We previously found that phosphorylation of two residues in the Icsbp DNA-binding domain increased affinity for the *GAS2* cis element. To determine the role of these residues in the influence of Shp2 on Icsbp-induced *GAS2* repression, we co-transfected U937 cells with the *GAS2* promoter/reporter construct, a vector to express Icsbp with mutation of the DNA-binding domain residues (Ymut-Icsbp) and vectors to express Bcr-abl, C463S-Shp2 or both. We found Ymut-Icsbp was significantly less efficient at *GAS2* repression in comparison to Wt Icsbp, with or without Bcr-abl (p<0.01, n=4), as anticipated [16]. Additionally, we found inhibition of Shp2 did not influence repression activity of Ymut-Icsbp in this assay (p>0.2, n=4 for Ymut-Icsbp + Bcr-abl with versus without C463S-Shp2) (Figure 1A). These results suggested Shp2 exerted an influence specifically through these Icsbp tyrosine residues. Bcr-abl, C463S-Shp2 and Icsbp vectors did not alter activity of the empty reporter vector and this value was subtracted as background.

Since Gas2 is a Calpain inhibitor, we investigated effects of Bcr-abl and Shp2 on Calpain activity. For these studies, we transduced primary myeloid progenitor cells from murine bone marrow with retroviral vectors to express Bcr-abl, C463S-Shp2, both, or control vector. Granulocyte-monocyte progenitor cells were used for these studies as these represent the CML-LSC in murine models (see Materials and Methods). We found significantly less Calpain activity in Bcr-abl^+^ cells versus control cells, consistent with impaired transcriptional repression of *GAS2* by Icsbp leading to Calpain inhibition (Figure 1B). Also consistent with our proposed mechanism, Calpain activity was significantly greater in cells co-expressing Bcr-abl plus C463S-Shp2 versus Bcr-abl alone (p<0.0001, n=3) (Figure 1B).

We also investigated the impact of Shp2 inhibition on tyrosine phosphorylation of Icsbp in Bcr-abl-transduced murine myeloid progenitor cells. For these experiments, cells were transduced with a vector to express Bcr-abl alone or with C463S-Shp2. Cell lysates were analyzed by immuno-precipitation with an anti-phospho-tyrosine antibody (under denaturing conditions) followed by Western blot with an Icsbp antibody. We found DN-Shp2 expression increased the abundance of tyrosine phosphorylated Icsbp in Bcr-abl^+^ cells (Figure 1C). Blots were also probed with a Gapdh antibody to indicate equivalent loading in the 1/10 input control lane. These results supported our hypothesis that Bcr-abl decreased Calpain activity through Shp2-related inhibition of *GAS2* repression by Icsbp. Interestingly, total Icsbp protein was also increased by Shp2-inhibition, as is explored in detail below.

**Figure 2:**
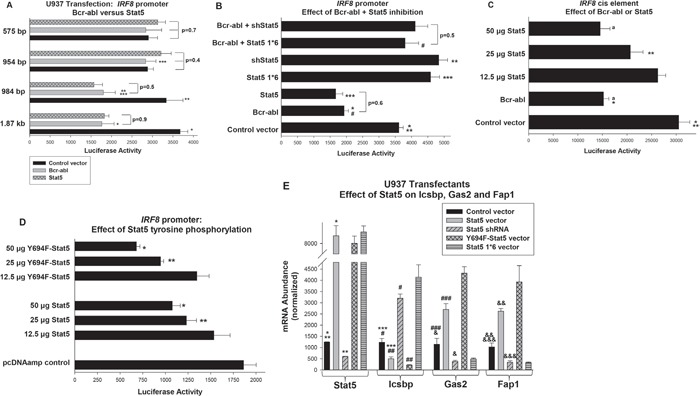
Decreased activity of the human *IRF8* promoter in Bcr-abl expressing cells did not require Stat5 tyrosine phosphorylation, but did require an NCoR-interaction domain in Stat5 **A.** Bcr-abl and Stat5 decreased activity of a cis element located between -984 and -954 bp in the human *IRF8* promoter. U937 cells were co-transfected with a series of reporter constructs representing truncations of the *IRF8* promoter and vectors to express Bcr-abl, Stat5 or control vector. Statistically significant differences indicated by *, **, or ***. **B.** Repression of the *IRF8* promoter by Bcr-abl required an NCoR-interaction domain in Stat5. U937 cells were co-transfected with the -984 bp *IRF8* promoter construct and combinations of vectors to express Bcr-abl, Stat5 specific shRNAs, Stat5 1*6 (NCoR-interaction domain mutant), or control vectors. Statistically significant differences indicated by *, ** *** or #. **C.** The Stat-binding consensus in the *IRF8* promoter functioned as a cis element. A construct with multiple copies of the -984 and -954 bp *IRF8* promoter sequence linked to a minimal promoter and reporter was co-transfected into U937 cells with vectors to express Bcr-abl or Stat5. Statistically significant differences in reporter activity indicated by * or **, and not significantly different activity by ‘a’. **D.** *IRF8* repression by Stat5 was impaired tyrosine phosphorylation of this transcription factor. U937 cells were co-transfected with the -984 bp *IRF8* promoter/reporter construct and increasing amounts of Stat5 or Y694F-Stat5 expression vectors (or control vector). Statistically significant differences indicated by * or **. **E.** Overexpression of Stat5 or Y694F-Stat5 decreases expression of endogenous Icsbp in U937 transfectants, but expression of Stat5 1*6 or Stat5 knockdown increases Icsbp expression. U937 cells were transfected with vectors to express WT or various mutants of Stat5 or a Stat5 specific shRNA expression vector (or relevant control vectors). Gene expression was quantified by real time PCR. Statistically significant differences indicated by *, **, ***, #, ##, ###, &, && or &&&. For all of these studies, p<0.02 was considered statistically significant.

In additional studies, we found the increased abundance of Gas2 protein in Bcr-abl^+^ murine bone marrow progenitor cells was reversed by expressing DN-Shp2 (Figure 1D). Expression of Gas2 mRNA was also significantly greater in cells expressing Bcr-abl alone versus Bcr-abl plus C463S-Shp2 (p<0.0001, n=6), and in Bcr-abl^+^ cells versus control cells (p<0.001, n=6) (Figure 1E). Icsbp mRNA was significantly less in Bcr-abl^+^ cells versus control cells, as anticipated [14, 16]. Decreased Icsbp mRNA in Bcr-abl^+^ cells was ameliorated by C463S-Shp2 (p=0.4, n=6 for comparison between control and Bcr-abl + C463S-Shp2) (Figure 2C), suggesting Shp2 influenced both tyrosine phosphorylation and mRNA expression of Icsbp.

Therefore, inhibition of Shp2 and increased calpain activity in Bcr-abl^+^ cells was correlated with increased Icsbp mRNA expression. These results suggested the possibility the *IRF8* promoter was repressed by a Calpain substrate. We next considered candidate Calpain substrates that might have this activity.

### Bcr-abl impairs transcription of the human IRF8 promoter in a Stat5 dependent manner

Since our prior studies identified βcatenin as a Calpain substrate relevant to the pathogenesis of CML, we first considered the possibility of *IRF8* transcriptional regulation by βcatenin [16]. However, these studies did not indicate *IRF8* transcriptional repression by this protein (not shown). Calpain has additional substrates implicated in CML pathogenesis, including Stat5. Stat5 activates some of its target genes and represses others. Stabilization of Stat5 protein in Bcr-abl^+^ cells would alter target gene transcription; increasing expression of activation targets, but decreasing expression of repression targets. Few of the latter have been identified, but other investigators defined a Stat5-binding cis element in the proximal murine *Irf8* promoter involved in transcriptional repression during differentiation/activation of a murine macrophage line [31].

Although the 5′ flanking regions in the human and murine genes encoding Icsbp are highly conserved, the transcription start sites (TSS) of the published promoters are different. In the murine *Irf8* gene, exon 1 is comprised of 52 bp of 5′ UTR [31]. The reported human exon 1 also is comprised of 5′ UTR, but is 421 bp in length [33]. Because of this difference, the sequence that is homologous to the reported Stat5-binding cis element in the murine *Irf8* promoter is in the 5′UTR of the human Icsbp message, not in the human *IRF8* promoter. Previously published studies of regulation of this gene were performed either with the murine *Irf8* promoter sequence, or with constructs containing human 5′ *IRF8* flank sequence, but using the murine TSS (i.e. including sequence 3′ to the human TSS) [31, 32]. Because of this discrepancy, we first determined if the reported human *IRF8* promoter was influenced by Bcr-abl in a Stat5-dependent manner.

We co-transfected U937 myeloid cells with a series of reporter constructs containing truncations of the reported human *IRF8* promoter and vectors to express Bcr-abl or Stat5 (or control vector). Either Bcr-abl or overexpressed Stat5 decreased activity of constructs with between 1.87 kb to 984 bp of *IRF8* promoter in comparison to control (p<0.0001, n=6) (Figure 2A). Bcr-abl and Stat5 did not influence activity of constructs with 954 bp or less, suggesting a repressor element between -984 and -954 bp in the human promoter (Figure 2A). We found no significant difference in maximal repression by Bcr-abl versus overexpressed Stat5 for constructs between 1.87 kb and 984 bp of the *IRF8* promoter (p=0.4, n=6). This sequence is conserved in the murine 5′ flank, but is just 5′ to sequences studied in prior *IRF8* promoter/reporter assays [32].

To determine if Bcr-abl was acting through Stat5, we tested the -984 bp *IRF8* promoter construct in transfectants with Bcr-abl and a vector to express Stat5-specific shRNAs (or scrambled shRNA control), or a form of Stat5 with mutation of a Nuclear Co-Repressor (NCoR)-interaction domain (the previously described Stat5 1*6). Stat5 1*6 is constitutively active for transcriptional activation targets, but would be anticipated to lack repression activity [34]. We found that either Stat5 knockdown or Stat5 1*6 expression increased *IRF8* promoter activity and reversed Bcr-abl-induced repression (p<0.0001, n=6 for reporter activity in Bcr-abl transfectants with versus without Stat5 shRNA or Stat5 1*6) (Figure 2B).

Between -985 and -954 bp, the *IRF8* promoter includes a Stat-binding consensus sequences (determined by Vista analysis) [35]. To test this sequence for repression activity, we generated a -985 bp *IRF8* promoter construct with mutation of the Stat-binding consensus. This mutant construct was co-transfected into U937 cells with vectors to express Bcr-abl, Stat5 or control vector. We found that activity of the mutant *IRF8* promoter construct was not altered by Bcr-abl or overexpressed Stat5 (p=0.6, n=4 for comparison of the three groups on the mutant promoter) (not shown).

As another method to test the function of this region as a cis element, we generated a reporter construct with three copies of the -985 to -954 bp sequence linked to a minimal promoter. This construct was transfected into U937 cells with vectors to express Bcr-abl, a dose titration of Stat5 or control vector (Figure 2C). We found that either Bcr-abl or Stat5 significantly decreased activity of the -985 to -954 bp *IRF8* construct (p<0.001, n=6), but had no effect on the minimal promoter/reporter control vector (subtracted as background). The effects of Bcr-abl or Stat5-overexpression on activity of this cis element and on the full -985 bp *IRF8* promoter construct were not significantly different (decrease with Bcr-abl 48.8% ± 5.3% versus 46.0% ± 6.1%, p=0.2 n=10; with Stat5 51.1% ± 13.6% versus 49.5% ± 3.2%, p=0.5, n=10). This suggested there were not additional distal Stat5 binding cis elements in the human *IRF8* promoter.

Prior studies determined phosphorylation of Stat5 tyrosine 694 was required for target gene activation [36]. To determine if phosphorylation of this residue was required for *IRF8* repression by Stat5, we co-transfected U937 cells with dose titration of vector to express Wt Stat5 or Y694F-Stat5. We found Y964F-Stat5 repressed the *IRF8* promoter significantly more efficiently than WT Stat5 (p<0.01, n=4 between 25 and 50 μg) (Figure 2D). These proteins are equivalently overexpressed in U937 cells under these conditions (not shown).

We next performed studies to correlate these reporter assays with effects of Stat5 on endogenous *IRF8* gene expression. For these studies, we analyzed expression U937 transfectants with vectors to express Stat5, Stat5 1*6, Y694F-Stat5 or a Stat5 specific shRNA (or relevant controls), as above. The effect on Icsbp expression was quantified by real time PCR. We found Stat5 overexpression significantly decreased Icsbp mRNA in comparison to empty vector (p<0.01, n=3) (Figure 2E), consistent with the reporter assays above. Overexpression of Y694F-Stat5 had also decreased Icsbp mRNA, but more efficiently than WT Stat5 (p<0.01, n=3). Conversely, either Stat5 1*6 or Stat5-knockdown significantly increased Icsbp mRNA (p<0.001, n=3). To determine the functional significance of altered Icsbp expression, we quantified expression of the Icsbp target genes encoding Gas2 and Fap1. We found increased expression of Fap1 and Gas2 in transfectants with Stat5 or Y694F-Stat5 (p<0.0001, n=3 for comparison to control cells), but decreased Fap1 and Gas2 in cells with Stat5 1*6 or Stat5-knockdown (p<0.001, n=3) (Figure 2E).

### Decreased IRF8 transcription in Bcr-abl expressing cells requires Shp2 activity and Calpain inhibition

**Figure 3:**
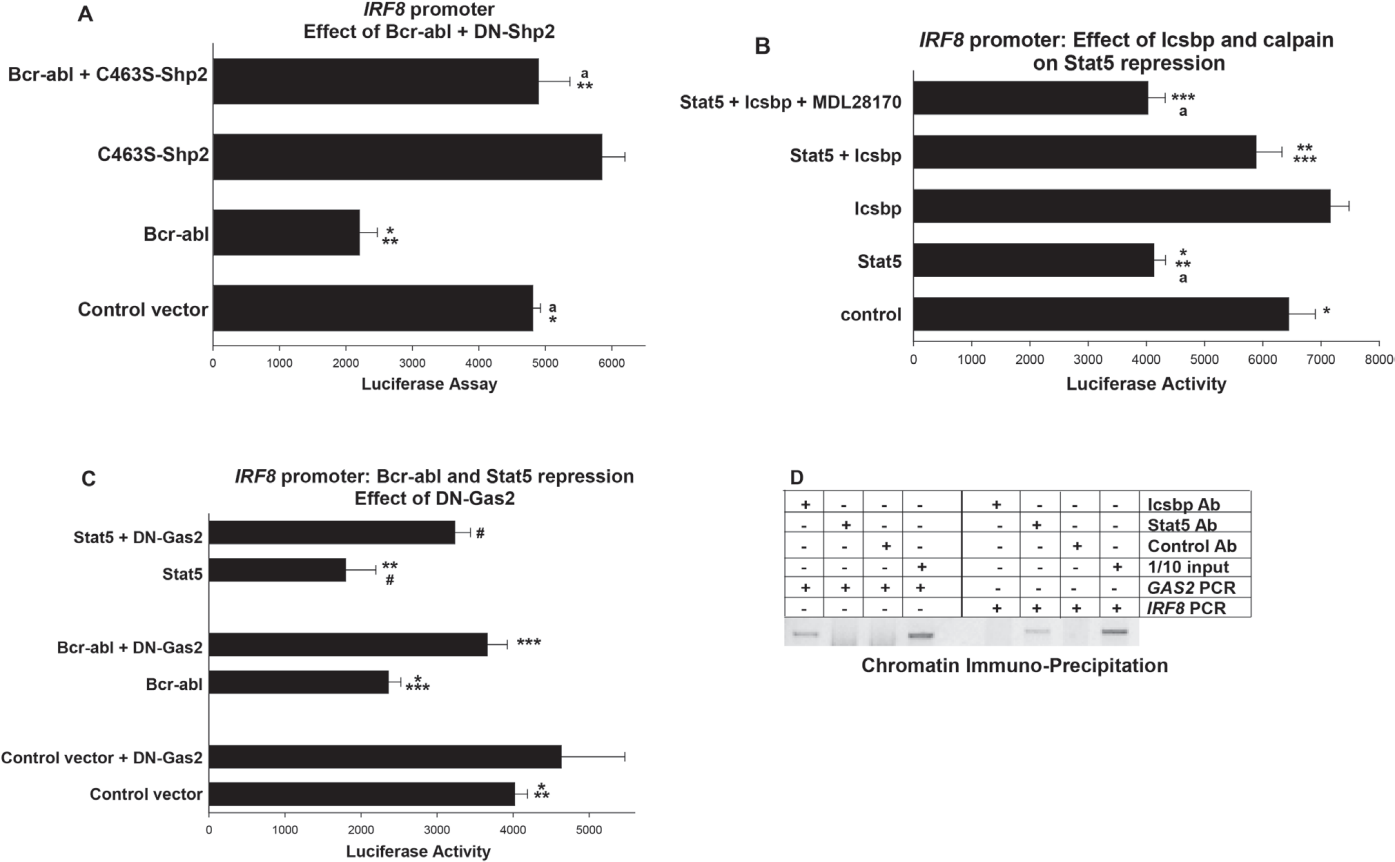
Repression of *IRF8* promoter activity by Bcr-abl and Stat5 required inhibition of Calpain by Gas2 U937 cells were transfected with a -984 bp of *IRF8* promoter/reporter construct. Transfectants were analyzed for effects of Shp2, Icsbp, Gas2 or Calpain on repression of the *IRF8* promoter by Bcr-abl/Stat5. **A.** Shp2 activity was required for repression of *IRF8* promoter activity by Bcr-abl. Cells were co-transfected with vectors to express combinations of Bcr-abl, C463S-Shp2, both, or control vectors. Statistically significant differences indicated by * or **, and not significantly different activity by ‘a’. **B.** Repression of the *IRF8* promoter by Stat5 was impaired by overexpression of Icsbp, but the Icsbp effect was reversed by calpain inhibition. Cells were co-transfected with combinations of vectors to express Stat5 or Icsbp, with or without a calpain inhibitor (MDL28170). Statistically significant differences indicated by *, ** or ***, and not significantly different activity by ‘a’. **C.** Inhibition of Gas2 impaired repression of the *IRF8* promoter by Bcr-abl or Stat5. Cells were co-transfected with vectors to express a dominant negative form of Gas2 (DN-Gas2) and Bcr-abl, Stat5 or control vector. Statistically significant differences indicated by *, **, *** or #. **D.** Stat5 bound the *IRF8* promoter *in vivo*, but Icsbp did not. Chromatin immuno-precipitation was performed with U937 cells and antibodies to Stat5, Icsbp or irrelevant control. Co-precipitating chromatin was amplified with primers to the *IRF8* promoter, *GAS2* promoter or *IRF8* intron1 (~1.0 kb each). Amplified DNA was separated on an agarose gel and bands visualized by ethidium bromide staining (negative image presented). Irrelevant antibody was a negative control and 1/10 input (non-precipitated) chromatin was a positive control. For all of these studies, p<0.02 was considered statistically significant.

We were next interested in determining if Bcr-abl regulated *IRF8* promoter activity by stabilizing Stat5 protein in a Shp2, Icsbp, Gas2 and Calpain dependent manner. As a first step in this process, we investigated the contribution of Shp2 to Bcr-abl-related *IRF8* repression. For these studies, we co-transfected U937 cells with a reporter construct with -984 bp of the human *IRF8* promoter and vectors to express Bcr-abl, C463S-Shp2, both, or control vector. We found inhibition of Shp2 rescued *IRF8* promoter activity in Bcr-abl-transfectants (p<0.001, n=6 for comparison of Bcr-abl transfectants with versus without C463S-Shp2) (Figure 3A). We hypothesized that increased tyrosine phosphorylation of Icsbp in Bcr-abl^+^ cells with Shp2 inhibition decreased Gas2 expression, with consequently increased Calpain activity and degradation of Stat5.

To investigate this mechanism, we studied the effect of Icsbp on *IRF8* promoter activity in Bcr-abl-transfectants. If our hypothesis is correct, Icsbp should impair Stat5 protein stability (by increasing Calpain activity) and thereby impair Stat5-induced repression of the *IRF8* promoter. We tested this by co-transfecting U937 cells with the -985 bp *IRF8* promoter/reporter construct and vectors to overexpress various combinations of Stat5 and Icsbp (or relevant control vectors). Icsbp-overexpression modestly increased *IRF8* promoter activity at high doses (not shown), so we chose an amount of Icsbp vector that alone had little effect on this construct. We found Icsbp overexpression significantly impaired Stat5-mediated repression of the *IRF8* promoter (p<0.001, n=4 for Stat5 with versus without Icsbp) (Figure 3B). To connect impaired Stat5 repression activity to increased Calpain activity in Icsbp-overexpressing transfectants, we treated some cells with a Calpain inhibitor (MDL28170). We found Calpain inhibition reversed the effect of Icsbp on Stat5-mediated repression of the *IRF8* promoter (p<0.01, n=4 for Stat5 + Icsbp with versus without Calpain inhibitor) (Figure 3B). *IRF8* promoter activity was not significantly different in transfectants with Stat5 alone versus Stat5 + Icsbp + Calpain inhibitor (p=0.7, n=4).

We next investigated the role of Gas2 in Bcr-abl-induced repression of *IRF8* transcription. For these studies, we co-transfected U937 cells with the -985 bp *IRF8* promoter-reporter construct and vectors to express Bcr-abl, a dominant negative form of Gas2 (DN-Gas2), both, or control vectors. This dominant negative form of Gas2 binds to, but does not inhibit, Calpain [24]. By preventing interaction of Calpain with endogenous Gas2, this DN-Gas2 effectively increases Calpain activity, as was elegantly demonstrated by other investigators [24].

Since we found overexpression of DN-Gas2 at high levels modestly increased *IRF8* promoter activity (not shown), we employed an amount of DN-Gas2 expression vector that did not alone have any effect on this promoter. Consistent with our hypothesis, we found that Calpain-activation (through inhibiting Gas2 with the DN form) increased activity of the *IRF8* promoter in Bcr-abl-transfectants (p<0.001, n=6 for Bcr-abl with versus without DN-Gas2). To connect this effect to Stat5, we co-transfected cells with the *IRF8* promoter construct and vectors to overexpress Stat5 with or without DN-Gas2 (or control vectors). We found that inhibiting Gas2 also blocked *IRF8* repression by overexpressed Stat5 (p<0.01, n= 6 for Stat5 with versus without DN-Gas2) (Figure 3C).

To determine if Stat5 exerted these effects through direct interaction with the *IRF8* promoter, we performed by chromatin immune-precipitation assays. For these experiments, we analyzed co-precipitating chromatin from U937 cell lysates using an antibody to Stat5 (versus irrelevant control antibody). Chromatin was amplified by PCR with primers flanking the proximal 1.0 kb of the human *IRF8* promoter (i.e. not including sequence homologous to the previously described Stat5 binding site in the murine promoter). Since overexpressed Icsbp also influenced *IRF8* promoter activity, we also performed control studies with an Icsbp antibody. Primers to the *GAS2* promoter (1.0 kb) or *IRF8* intron 1 were negative controls. We found specific co-precipitation of the *IRF8* promoter with Stat5, but not with intron sequence from this gene or with the *GAS2* promoter (Figure 3D). Conversely, Icsbp co-immuno-precipitated the *GAS2* promoter, but not the *IRF8* promoter.

This study was repeated using DNA from two independent co-immuno-precipitation experiments and a representative image is shown. The goal was to verify binding of Stat5 to this region of the human *IRF8* promoter and establish Icsbp did not interact with this region of the promoter. Also, to demonstrate Icsbp, but not Stat5, bound the *GAS2* promoter region under investigation. Amplified co-immuno-precipitating DNA was quantified using scanned images. We found the abundance of *GAS2* promoter DNA co-precipitating with Stat5 antibody was not significantly different than the amount co-precipitating with control antibody (p>0.4, n=6 for two images in triplicate). However, Icsbp antibody co-precipitated significantly more *GAS2* promoter DNA in comparison to control antibody (p<0.001, n=6). Conversely, the amount of *IRF8* promoter DNA that co-immuno-precipitated with antibody to Icsbp was not significantly different than with control antibody (p>0.6, n=6). Antibody to Stat5 co-immuno-precipitated significantly more *IRF8* promoter than control (p<0.001, n=6).

### Loss of Icsbp increased Stat5 protein by increasing Gas2 expression and decreasing Calpain activity

To make the functional connection between Bcr-abl, decreased Calpain activity and decreased degradation of Stat5, we first investigated the influence of Icsbp and Gas2/Calpain on Stat5. For these studies, we transduced murine bone marrow myeloid progenitor cells from Wt or Icsbp^−/−^ mice with a retroviral vector to express DN-Gas2 or control vector. Cells were analyzed for Calpain activity and expression of Stat5 protein and mRNA were quantified.

**Figure 4:**
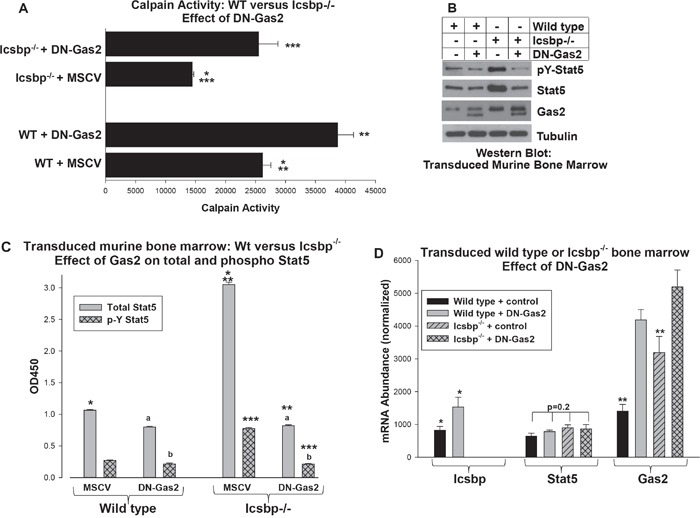
Increased Gas2 expression in Icsbp−/− myeloid progenitor cells decreased Calpain activity and increased Stat5 protein Wild type or Icsbp^−/−^ bone marrow-derived murine myeloid progenitor cells were transduced with a vector to express a dominant negative form of Gas2 (DN-Gas2) or control vector. **A.** Calpain activity was increased by Gas2 inhibition in Icsbp^−/−^ myeloid progenitor cells. Cells were analyzed for calpain activity. Statistically significant differences indicated by *, ** or ***. **B.** Inhibition of Gas2 in Icsbp^−/−^ myeloid progenitor cells decreased Stat5 protein. Cell lysates were analyzed for protein expression by serially probing Western blots with antibodies as indicated. **C.** Both total and phospho Stat5 protein were decreased by inhibition of Gas2 in Icsbp^−/−^ myeloid progenitor cells. Cell lysates were analyzed by ELISA for total and pY694-Stat5. Statistically significant differences indicated by *, ** or ***, and not significantly different values by ‘a’ or ‘b’. **D.** Stat5 mRNA expression was not altered by Icsbp-knockout or Gas2 inhibition, but Gas2 inhibition increased Icsbp mRNA in Wt cells. Expression of mRNA for Stat5, Gas2 and Icsbp was determined by real time PCR. Statistically significant differences indicated by * or **. For all of these studies, p<0.02 was considered statistically significant.

We found significantly less Calpain activity in Icsbp^−/−^ myeloid progenitor cells versus Wt control cells (p<0.001, n=3), but this was reversed by expression of DN-Gas2 (p<0.0001, n=3 for Icsbp^−/−^ cells with versus without DN-Gas2) (Figure 4A). We found total and phospho Stat5 protein were both more abundant in Icsbp^−/−^ myeloid progenitors versus control cells, but this difference was abolished by DN-Gas2 (Figure 4B). To quantify total and tyrosine phosphorylated Stat5 in these transduced cells, we performed enzyme linked immune-absorbance assays (ELISA) (Figure 4C). Consistent with the Western blots, total Stat5 protein was greater in Icsbp^−/−^ myeloid progenitor cells versus Wt cells (p<0.0001, n=3). Expression of DN-Gas2 decreased Stat5 protein in these cells so that it was not significantly different than in DN-Gas2-expressing Wt cells (p=0.6, n=3). The absolute amount of phospho-Stat5 (pY694) was also significantly greater in Icsbp^−/−^ versus control myeloid progenitors (Figure 4C), but the ratio of phospho/total Stat5 was not significantly different in Wt versus Icsbp^−/−^ cells (p=0.20, n=3). Expression of DN-Gas2 significantly decreased the absolute amount of phospho-Stat5 in both Icsbp^−/−^ and Wt cells (p<0.001, n=3). However, DN-Gas2 did not significantly alter the ratio of phospho/total Stat5 in either Wt or Icsbp^−/−^ cells.

Consistent with our hypothesis, neither Icsbp-knockout nor DN-Gas2 altered Stat5 mRNA expression in Wt or Icsbp^−/−^ bone marrow progenitor cells (Figure 4D). In control experiments, we confirmed expression of DN-Gas2 in transduced cells (Figure 4B and 4D). We also found increased Icsbp mRNA in Wt cells expressing DN-Gas2 (Figure 4D), consistent with decreased Stat5 protein in these cells (Figure 4B and 4C). Calpain protein and mRNA expression are not altered by Icsbp loss or Gas2-inhibition [16]. This supported our hypothesis that Icsbp destabilized Stat5 protein in a Gas2/Calpain-dependent manner.

### Bcr-abl increased Stat5 protein by increasing Gas2 expression and decreasing Calpain activity

**Figure 5:**
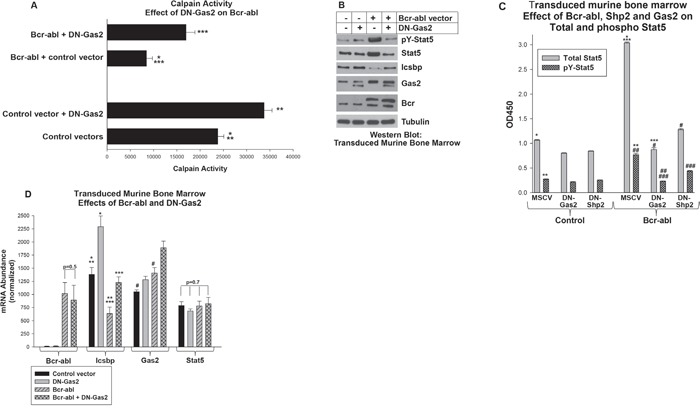
Increased Stat5 protein in Bcr-abl+ myeloid progenitor cells required Calpain inhibition by Gas2 Murine myeloid progenitor cells were transduced a Bcr-abl retroviral expression vector with or without vectors to express dominant negative forms of Gas2 (DN-Gas2) or Shp2 (C463S-Shp2) or control vectors. **A.** Calpain activity in Bcr-abl^+^ myeloid progenitor cells was increased by inhibition of Gas2. Transduced cells were analyzed for Calpain activity. Statistically significant differences indicated by *, ** or ***. **B.** Inhibition of Gas2 in Bcr-abl^+^ myeloid progenitor cells decreased Stat5 protein, but increased Icsbp protein. Cell lysates were analyzed for protein expression by serially probing Western blots with antibodies as indicated. **C.** Total and phospho Stat5 were equivalently decreased by Gas2 inhibition in Bcr-abl^+^ myeloid progenitor cells. Inhibition of Shp2 also decreased total and phospho Stat5 in these cells, but increased the pY/total ratio. Cell lysates were analyzed by ELISA for total and pY694-Stat5. Statistically significant differences indicated by *, **, ***, #, ## or ###. **D.** Inhibition of Gas2 in Bcr-abl^+^ cells increased Icsbp mRNA, but did not alter Stat5 mRNA expression. Expression of Bcr-abl, Icsbp, Gas2 and Stat5 was determined by real time PCR. Statistically significant differences indicated by *, **, *** or #. For all of these studies, p<0.02 was considered statistically significant.

We next investigated the role of Bcr-abl, Gas2 and Calpain on Stat5 protein expression in transduced myeloid progenitor cells. In initial studies, murine bone marrow-derived myeloid progenitor cells were transduced with retroviral vectors to express Bcr-abl with or without DN-Gas2 (or with control vectors) and Calpain activity was determined (Figure 5A). We found significantly less Calpain activity in Bcr-abl-transduced progenitor cells versus control cells (p<0.001, n=3), but this difference was largely resolved by expression of DN-Gas2.

To determine the impact of Calpain activation on Stat5 protein, we quantified total and phospho Stat5 in transduced cells. Abundance of both was increased in Bcr-abl-transduced cells in comparison to control cells (Figure 5B). By ELISA, we determined Bcr-abl significantly increased total Stat5 protein in comparison to cells with control vector (p<0.0001, n=3, approximately 3 fold greater) (Figure 5C). This was also reflected in a proportionate increase in phospho Stat5 in Bcr-abl transduced versus control cells.

Expression of DN-Gas2 decreased both total and phospho Stat5, consistent with degradation of Stat5 by Calpain in these cells (Figure 5B). DN-Gas2 decreased total Stat5 protein in both control and Bcr-abl cells, but the absolute decrease was significantly greater in Bcr-abl^+^ cells (p<0.001, n=3) (Figure 5C). The amount of total Stat5 in control cells with DN-Gas2 versus Bcr-abl + DN-Gas2 was not significantly different (p=0.8, n=3). Phospho Stat5 decreased proportionately to total Stat5 in both Bcr-abl-transduced and control cells upon DN-Gas2 expression (Figure 5C). Control blots demonstrated expression of the DN-Gas2 in transduced cells, but also demonstrated increased expression of endogenous Gas2 in Bcr-abl transduced cells relative to control (Figure 5B).

In contrast to protein expression results, total Stat5 mRNA was not altered by expression of either Bcr-abl or DN-Gas2 (Figure 5D). However, Icsbp mRNA was significantly increased by expression of DN-Gas2 in Bcr-abl^+^ progenitor cells and control cells (p<0.0001, n=3) (Figure 5D). This was consistent with increased *IRF8* promoter activity, due to decreased Stat5 protein, in cells with Calpain activation (i.e. inhibition of a Calpain inhibitor).

### Bcr-abl-induced increase in Stat5 protein required Shp2 activity

In the first section of this work, we found expression of DN-Shp2 increased Calpain activity in Bcr-abl transduced murine myeloid progenitor cells (Figure 1B). In this section, we followed up on this observation by investigating the impact of DN-Shp2 on Stat5 protein in murine bone marrow derived myeloid progenitor cells transduced with a vector to express Bcr-abl (or control vector) with or without expression of C463S-Shp2. In Western blots of lysate proteins, we found DN-Shp2 decreased total and phospho Stat5 in Bcr-abl transduced myeloid progenitor cells (Figure 1D). We quantified these results by ELISA assays for phospho and total Stat5, as described above (Figure 5C). In these assays, although C463S-Shp2 decreased both total and phospho Stat5 in Bcr-abl transduced cells, the ratio did not remain constant as it did upon expression of DN-Gas2 in Bcr-abl^+^ cells. Instead, the ratio of phospho to total Stat5 was significantly increased by inhibition of Shp2, consistent with Stat5 as an Shp2 substrate in these cells (~23% phospho/total for Bcr-abl, but 34% phospho/total for Bcr-abl + DN-Shp2) (Figure 5C).

### Shp2 and Gas2 regulate calpain activity and Icsbp expression in primary human CML cells

**Figure 6:**
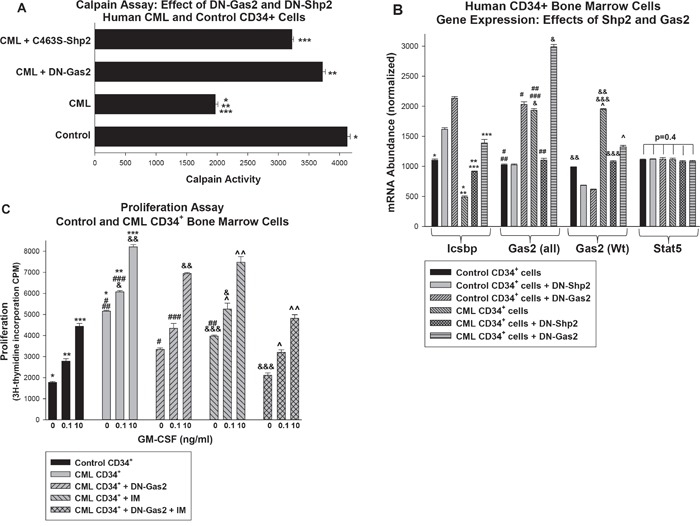
Inhibition of Shp2 or Gas2 increased Calpain activity and Icsbp expression in primary human CD34+ bone marrow cells from CML subjects Bone marrow CD34^+^ progenitor cells were isolated from CML or control human subjects. Some cells were transduced with retroviral vectors to express dominant negative forms of Gas2 (DN-Gas2) or Shp2 (C463S-Shp2) (or with control vector). **A.** Calpain activity was relatively decreased in human CML CD34^+^ cells versus control, but this was reversed by inhibition of Gas2 or Shp2. Cell lysates were analyzed for Calpain activity. Statistically significant differences indicated by *, ** or ***. **B.** Inhibition of Gas2 or Shp2 increased Icsbp mRNA in human CD34^+^ CML cells, but did not alter Stat5 mRNA expression. Cell lysates were analyzed for mRNA expression of Icsbp, Gas2 or Stat5 by real time PCR. These studies used two sets of Gas2 PCR primers; one that did (Gas2-all) and one that did not (Gas2) recognize the dominant negative form. Statistically significant differences indicated by *, **, ***, #, ##, ###, &, &&, &&& or ^. **C.** Inhibiting Gas2 increased the effect of imatinib on proliferation in CD34^+^ CML cells. Some cells described above were analyzed for proliferation in response to a dose titration of GM-CSF. Statistically significant differences in by 3H-thymidine incorporation at a given cytokine dose are indicated by *, **, ***, #, ##, ###, &, &&, &&&, ^ or ^^. For all of these studies, p<0.02 was considered statistically significant.

We also investigated the roles of Shp2 and Gas2 in the more molecularly complex setting of human CML. For these studies, CD34^+^ cells from the bone marrow of CML subjects were compared to CD34^+^ bone marrow cells from normal subjects. CML cells were transduced with retroviral vectors to express DN-Shp2 or DN Gas2 and analyzed for calpain activity (Figure 6A). We found significantly less Calpain activity in CML versus control bone marrow cells (p<0.0001, n=3), but this difference was almost completely reversed by either C463S-Shp2 or DN-Gas2. This was consistent with our result in transduced murine bone marrow cells and suggested this was a reasonable model to explore the biology of CML.

We analyzed expression of Icsbp and Gas2 in these cells to determine if either Shp2 inhibition or Calpain activation might be a rationale therapeutic approach to CML (Figure 6B). We found significantly less Icsbp expression in CD34^+^ CML cells versus control (p<0.0001, n=3), but Icsbp expression was significantly increased in these cells by DN-Shp2 or DN-Gas2 (p<0.001, n=3). We analyzed expression of Gas2 in these cells using two sets of primers; one that recognized both endogenous Gas2 and the DN-Gas2 recombinant protein (referred to Gas2 all) and one that recognized only endogenous Gas2 (referred to as Gas2) (Figure 6B). We found significantly more expression of endogenous Gas2 in CD34^+^ CML cells versus control cells (p<0.0001, n=3), but inhibition of either Shp2 or Gas2 almost completely reversed this Bcr-abl effect (Figure 6B).

We further analyzed the transduced cells to determine effects of Gas2/Calpain on proliferation. For these studies, cells were deprived of cytokines for 24 hours followed by treatment with a dose titration of GM-CSF for 24 hours to stimulate proliferation. Some cells were also treated with imatinib (IM) during this final 24 hour period. We found significantly greater baseline and cytokine stimulated proliferation in CML CD34^+^ cells in comparison to control CD34^+^ cells (p<0.001, n=3) (Figure 6C). Proliferation was decreased significantly by DN-Gas2 at all cytokine doses (p<0.001, n=3) and by IM at the lower doses (p<0.002, n=3 for 0 and 0.1 ng/ml; p=0.03, n=3 for 10 ng/ml). Adding DN-Gas2 to IM significantly decreased proliferation in comparison to IM alone (p<0.001, n=3 for all cytokine doses). We found proliferation was not significantly different in control CD34^+^ cells in comparison to CD34^+^ CML cells with DN-Gas2 plus IM (p>0.04, n=3 for all three cytokine doses). In contrast, there was a statistically significant difference in proliferation of CD34^+^ control cells in comparison to CD34^+^ CML cells treated with IM or DN-Gas2 alone (p<0.001, n=3 comparing either condition to control cells at each cytokine dose). This identified a functional effect of adding Calpain activation to tyrosine kinase inhibition in CML CD34^+^ cells.

## DISCUSSION

**Figure 7:**
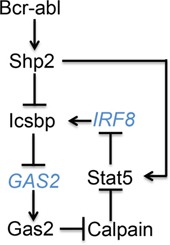
Graphical depiction of cross regulation between Icsbp and Stat5 in Bcr-abl+ progenitor cells Black letters represent proteins and blue letters represent genes.

Expression of Icsbp is decreased in human CML and re-expression of Icsbp in Bcr-abl^+^ bone marrow delays progression of CML in a murine transplant model [10, 11]. These data suggest rational therapeutic approaches to human CML might include increasing Icsbp expression. Conversely, increased expression of phosphorylated Stat5 was described in human CML cells and Stat5-knockout delays CML-development in murine models [37, 38]. These data suggest decreasing Stat5 protein might also be a productive therapeutic strategy for CML. In the current work, we found activation of Shp2 by Bcr-abl decreased Icsbp tyrosine phosphorylation, which subsequently decreased repression of *GAS2* transcription by Icsbp. Increased expression of Gas2, a Calpain inhibitor, stabilized Stat5 protein; a Calpain substrate. Increased Stat5 secondarily decreased *IRF8* transcription through a Stat5-binding negative cis element. In brief, our study identified a novel feedback mechanism between Icsbp and Stat5 in CML that potentially increased pro-leukemia effects of Stat5 and decreased leukemia suppression by Icsbp (Figure 7).

We have documented stabilization of at least two Calpain substrates relevant to the pathogenesis of CML in Bcr-abl^+^ cells; βcatenin (previously shown) and Stat5 (in this work) [16]. Our results also suggested a role for this feedback loop in normal myelopoiesis due to decreasing Gas2 expression, caused by increasing tyrosine phosphorylation of Icsbp, as differentiation proceeds. This would increase Calpain activity and favor degradation of Stat5, impairing expression of Stat5-activation-targets involved in cell proliferation and survival in differentiating granulocytes. Conversely, increased Stat5 in myeloid progenitor cells would favor expansion of this population through these activation targets and by repressing *IRF8* transcription. We found the latter does not require cytokine induced Stat5 tyrosine phosphorylation, so could occur in the absence of differentiating signals.

Our studies suggested an under-appreciated role for Calpain in the biology of CML. There are several additional Calpain substrates that might be relevant to CML pathogenesis, including Stat3 and Xiap1 [26, 39]. We investigated Stat3 for activity on the human *IRF8* promoter, but did not see an effect of either overexpression or knockdown of this transcription factor (data not shown). Xiap1 is a Calpain substrate of potential interest, since our previous studies identified regulation of Fas-induced apoptosis as a role for Icsbp during myelopoiesis and in leukemogenesis [14, 15, 23].

We chose to investigate the impact of Stat5 on the human promoter (as reported in ENSEMBL data base), rather than the human sequence with homology to the previously described murine promoter used by other investigators in prior studies [32]. The 5′ flanks of the murine and human genes are highly conserved, so differences in promoter usage in the mouse versus human are not sequence driven. It is possible different promoters are used for *IRF8* regulation under various conditions or in diverse lineages. This possibility is supported by the fact that the murine *Irf8* promoter was defined using a murine monocyte/macrophage cell lines undergoing dendritic-like differentiation/activation by inflammatory mediators [31].

Alternative promoter use under various conditions is consistent with our studies of the *NCF2* promoter (encoding the p67^phox^ phagocyte oxidase protein). We found *NCF2* promoter usage differed at steady state versus during monocytoid-differentiation with inflammatory cytokines [40, 41]. We similarly hypothesize the reported human *IRF8* promoter regulates expression of Icsbp during myelopoiesis and the reported murine promoter modulates expression during activation of inflammatory cells, or differentiation to subsets of effectors (such as dendritic cells). For example, the proximal Stat-binding cis element described in the murine promoter may be most effectively modulated by Stats during activation of mature inflammatory cells and the distal cis element, described for the first time in our work, by Stat5 during myelopoiesis. This is a subject of ongoing study in the laboratory.

Phosphorylation of a specific Stat5 tyrosine residue is required for activation of previously described target genes [36]. In the current studies, we found mutation of this Stat5 residue increased efficiency of *IRF8* promoter in comparison to wild type Stat5 protein. This observation is consistent with a role for Stat5 in repressing *IRF8* transcription in immature myeloid progenitor cells, but with decreasing activity during myelopoiesis due to cytokine-induced Stat5 phosphorylation. These results suggested Stat5 activity is modulated both by post translational modification and by protein stabilization/destabilization in a dynamic manner during hematopoiesis. Stat5 mRNA expression was not altered by Bcr-abl expression, Icsbp-knockout, or Shp2-inhibiton. In contrast, Icsbp activity was regulated during myelopoiesis by post translational modification and transcriptional modulation of the *IRF8* gene, but we have not identified conditions that favor degradation of Icsbp protein.

Our studies suggest Icsbp influences multiple mechanisms contributing to regulation of hematopoiesis and leukemogenesis, including regulation of protein stability. Our findings also indicate activity of Stat5 is not only regulated by mRNA expression and phosphorylation events, but also by protein stability. Such an integrated approach may identify previously unsuspected candidates, such as Calpain, as targets for modulation to address inflammatory and/or malignant disease states. Our studies also suggest Calpain activation as a potential therapeutic approach to augment the effects of tyrosine kinase inhibitors in CML.

## MATERIALS AND METHODS

### Protein expression vectors

The Icsbp/Irf8 cDNA was obtained from Dr. Ben Zion-Levi (Technion, Haifa, Israel), the full length cDNA was generated by PCR and subcloned into the mammalian expression vector pcDNA (Stratagene, La Jolla, CA), as described [40]. Bcr-abl (p210) cDNA in the MiGR1 retroviral vector were obtained from Dr. Ravi Bhatia (University of Alabama, Birmingham) and subcloned into the pcDNA expression vector. The cDNA for Gas2 was obtained from Invitrogen and cloned into the pcDNA and MSCV vectors, as described [16]. A dominant negative form of Gas2 (Δ171-314) was generated by PCR as described [16, 24]. Wild type and Y695F mutant murine Stat5 cDNAs were obtained from Addgene and subcloned in to the pcDNA and MSCV vectors. A form of Stat5 with mutation of the NCor-interaction domain (1*6-Stat5) was a gift from Dr. Jonathan Licht (University of Florida, Gainesville FL) and subcloned into pcDNAamp [34]. Wt and dominant negative (C463S) Shp2 cDNAs were a gift from Dr. Stuart Frank (University of Alabama, Birmingham) and were subcloned into the pcDNA and MSCV vectors. (Y92/94F)-Icsbp (Ymut-Icsbp) was generated and subcloned into pcDNAamp as described [30].

### Reporter constructs

The human *IRF8* 5′ flank (1.87 kb from the transcription start site) was generated by PCR from the U937 myeloid cell line. The genomic clone was sequenced to ensure identity with the sequence in the ENSEMBL data base [33]. This sequence and additional truncations of the *IRF8* promoter (−1.87 kb, −984 bp, −954 bp and −934 bp) were subcloned into the pGL3-basic reporter vector (Promega). Other constructs were generated with three copies of the *IRF8* −984 to −954 bp sequence subcloned into a minimal promoter-reporter vector (pGL3-promoter vector) (Promega). A reporter construct with the proximal 130 bp of *GAS2* promoter (containing an Icsbp binding cis element) in the pGL3-basic vector was previously described [16].

### Myeloid cell line culture

The human myelomonocytic leukemia cell line U937 [42] was obtained from Andrew Kraft (University of Arizona, Tucson). Cells were maintained as described [42].

### Murine bone marrow transduction

Animal studies were performed according to a protocol approved by the Animal Care and Use Committees of Northwestern University and Jesse Brown VA Medical Center. Bone marrow mononuclear cells were obtained from the femurs of Wt or Icsbp^−/−^ C57/BL6 mice. Granulocyte/monocyte progenitor cells were cultured (2 × 10^5^ cells per ml) in DME media supplemented with 10% fetal calf serum, 1% pen-strep, 10 ng/ml murine GM-CSF (R & D Systems Inc., Minneapolis, MN), 10 ng/ml murine recombinant IL-3 (R & D Systems Inc.) and 100 ng/ml of murine recombinant stem cell factor (Scf: R & D Systems Inc.) (referred to as myeloid progenitor cells in these studies). CD34^+^ cells were separated from the cultures using the Miltenyi magnetic bead system for extraction of RNA or proteins.** These cells represent the CML-LSC in murine models during CP [43]. For some experiments, cells were transduced with retroviral vectors to express p210 Bcr-abl (in MiGR1 vector), DN-Gas2 (in MSCVpuro vector), or DN-Shp2 (in MSCVneo vector). Replication incompetent retroviral vectors were generated using the Ecopack packaging plasmid, as described [23].

### Human bone marrow transduction

Human bone marrow cells from CML subjects were obtained under the auspices of approved protocols by the IRB of Northwestern University and Jesse Brown VA in accordance with an assurance filed with and approved by the U.S. Department of Health and Human Services. CD34^+^ cells were separated using the Miltenyi magnetic bead antibody affinity technique, as described for murine bone marrow cells. Cells were cryopreserved and compared under the same conditions as CD34^+^ normal human bone marrow cells purchased from Stem Cell Technologies (Vancouver, Canada). Cells were transduced as described for primary murine bone marrow cells, above.

### Quantitative real time PCR

RNA was isolated using the Triazol reagent (Gibco-BRL, Gaithersburg MD) and tested for integrity by denaturing gel electrophoresis. Primers were designed with software from Applied Biosystems and PCR performed using SYBR green according to the “standard curve” method. Result were normalized to 18S and actin to control for mRNA abundance.

### Transfectant of myeloid cell lines

For reporter gene assays, U937 cells (25 × 10^6^/ml) were transfected with various combinations of vectors to express p210 Bcr-abl, Wt or mutant Stat5, Wt or DN-Gas2, Wt or Ymut-Icsbp, Wt or DN-Gas2, or control (50 μg). Cells were co-transfected with the *IRF8* or *GAS2* promoter-reporter constructs (30 μg) and a CMV/renilla-luciferase reporter (control for transfection efficiency). Transfectants were assayed using Promega's Dual Luciferase Assay System (Promega, Madison, WI).

### Western blot and ELISA of lysates proteins

For Western blots, cells were lysed by boiling in 2X SDS sample buffer. Lysate proteins (50 μg) were separated by SDS-PAGE (8% acrylamide), transferred to nitrocellulose, and filters were serially probed with antibodies as described [13]. Each experiment was repeated at least three times with different sets of lysates and a representative blot is shown. In other experiments, total and tyrosine phosphorylated Stat5 protein in cell lysates were quantified by commercially available ELISA (Abcam, Cambridge, MA). ELISAs were performed in duplicate on three independent sets of lysates and results were graphed as OD450 (per manufacturer's instructions).

### Chromatin immunoprecipitation

Cells were incubated briefly in media supplemented with formaldehyde, lysates were sonicated to generate chromatin fragments with an average size of 1.0 kb, and immune-precipitated with Stat5, Icsbp, or irrelevant control antibody (Santa Cruz Biotechnology, Santa Cruz, CA) [14, 16]. Input chromatin (not precipitated) was a positive control. Chromatin was amplified by PCR with primers that amplified ~1.0 kb of the *GAS2* or *IRF8* promoter, or sequence from the first exon of the *IRF8* promoter (negative control). The experiment was repeated with two independent co-immuno-precipitations and scanned images were analyzed using Un-Scan-It Software (Silk Scientific, Orem, UT).

### Calpain assays

Calpain activity assays were performed using a commercially available kit (Biovision, Mountain View, CA). Cell lysates were assayed using the fluorescent labeled substrate provided in the kit and Calpain activity determined by flurometric change [16].

### Cell proliferation assays

CD34^+^ bone marrow cells were cultured in 10 ng/ml GM-CSF, 10 ng/ml IL3, 100 ng/ml SCF, deprived of cytokines for 24 hours (in DME with 10% FCS), and stimulated for 24 hours with a dose titration of GM-CSF (0.01 to 10 ng/ml). Imatinib (1.0 μg/ml) or buffer control was added for the last 24 hours. Proliferation was determined by adding 3H-thymidine for the last 8 hours of the assay.

### Statistical analysis

Statistical significance was determined by Student's t-test and ANOVA methods using the SigmaPlot and SigmaStat software. Results are reported as the average ± SEM and differences between samples are considered statistically significant if p<0.02.
